# Advances in understanding the role of gut microbiota in fat deposition and lipid metabolism

**DOI:** 10.1186/s40104-025-01284-9

**Published:** 2025-11-18

**Authors:** Yi Zhong, Yuhang Lei, Shan Jiang, Dujun Chen, Xinyi Wang, Kai Wang, Tianci Liao, Rongjie Liao, Mailin Gan, Lili Niu, Ye Zhao, Lei Chen, Xiaofeng Zhou, Yan Wang, Li Zhu, Linyuan Shen

**Affiliations:** 1 Farm Animal Genetic Resources Exploration and Innovation Key Laboratory of Sichuan Province, Sichuan Agricultural University, Chengdu, 611130 China; 2 State Key Laboratory of Swine and Poultry Breeding Industry, Sichuan Agricultural University, Chengdu, 611130 China; 3 Key Laboratory of Livestock and Poultry Multi-Omics, Ministry of Agriculture and Rural Affairs, College of Animal and Technology, Sichuan Agricultural University, Chengdu, 611130 China

**Keywords:** Bile acids, Fat deposition, Gut–brain axis, Gut–liver axis, Gut microbiota, Short-chain fatty acids

## Abstract

The gut microbiota has emerged as a pivotal regulator of host lipid metabolism and energy homeostasis. A growing body of evidence reveals that variations in the composition and metabolic activity of intestinal microbes are closely associated with differences in adipose tissue deposition across species. Notably, increased abundance of Firmicutes and a reduced proportion of Bacteroidetes and butyrate-producing bacteria have been linked to enhanced fat accumulation. Key microbial metabolites such as short-chain fatty acids (SCFAs) influence lipid metabolism through multiple pathways, including the activation of GPR41/43 receptors, modulation of the bile acid–FXR/TGR5 axis, and regulation of hepatic lipogenesis. Additionally, the gut–brain axis plays a critical role in controlling feeding behavior via neuroendocrine signaling. This review summarizes current advances in understanding the roles of dominant bacterial phyla and beneficial genera—including *Clostridium butyricum* and *Faecalibacterium prausnitzii*—in fat metabolism. We further explore the mechanisms by which gut microbiota modulate lipid synthesis and catabolism through SCFA production, bile acid signaling, and AMPK/PPAR-related pathways. These insights highlight the potential of microbiota-targeted strategies to restore lipid metabolic balance, offering novel opportunities for applications in health management, nutritional interventions, and microbial therapeutics.

**Graphical Abstract:**

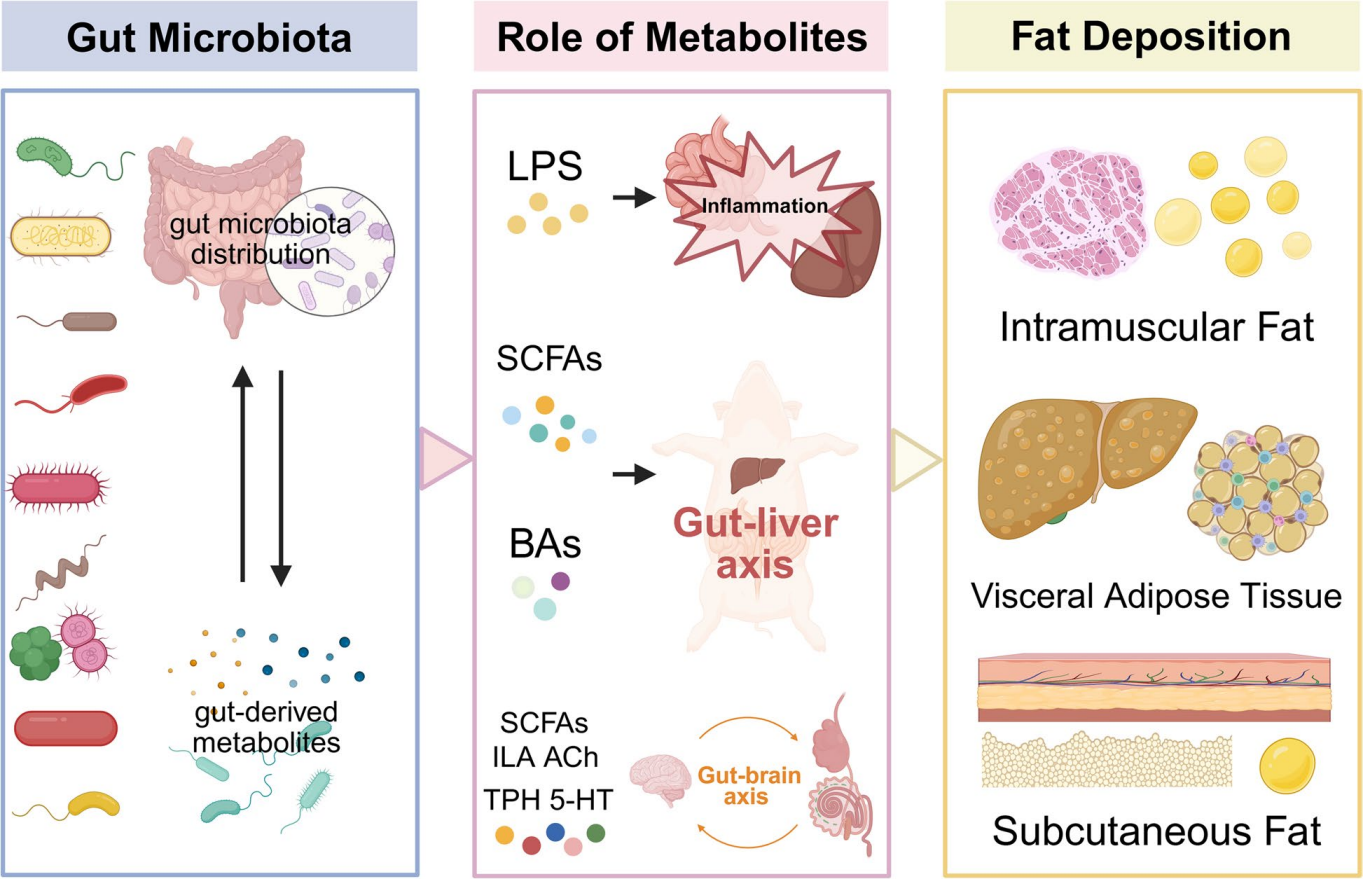

## Introduction

Fat deposition and lipid metabolism are fundamental physiological processes crucial for energy storage and utilization in livestock and poultry [1, 2]. In animal production, the regulation of these pathways is a primary determinant of economic efficiency and product quality [3]. Excessive deposition of abdominal and subcutaneous fat reduces feed conversion efficiency and carcass yield, representing a significant economic loss for producers [4]. Conversely, intramuscular fat (IMF), or marbling, is essential for meat palatability, directly enhancing tenderness, juiciness, and flavor, and is a key driver of consumer preference and premium pricing [5, 6]. Understanding the mechanisms that govern site-specific fat deposition is therefore critical for developing strategies to optimize carcass composition and improve meat quality.

The gastrointestinal tract harbors a vast and complex community of microorganisms, collectively known as the gut microbiota [7]. This dynamic ecosystem, comprising bacteria, fungi, viruses, and archaea, functions as a critical regulator of host physiology and metabolism [8]. Emerging research highlights the profound influence of the gut microbiota on host energy balance, including the regulation of fat deposition and systemic lipid metabolism [9]. Studies in swine and poultry demonstrate that distinct microbial profiles are associated with lean versus fat phenotypes, and these traits can be transmitted through fecal microbiota transplantation, underscoring a causal role of the microbiome in shaping carcass composition [10, 11].

The gut microbiota exerts its metabolic effects through multiple interconnected mechanisms. Firstly, variations in the composition and diversity of the gut microbial community are strongly associated with differential levels of fat accumulation and altered lipid profiles [12, 13]. Shifts in the relative abundance of dominant phyla, such as the Firmicutes-to-Bacteroidetes ratio, and the presence of specific bacterial genera have been linked to metabolic phenotypes [14, 15]. Secondly, the gut microbiota produces a diverse array of bioactive metabolites that act locally within the gut or enter systemic circulation to influence distant organs [16]. Key microbial metabolites implicated in lipid metabolism include short-chain fatty acids (SCFAs), such as acetate, propionate, and butyrate, generated from the fermentation of dietary fibers, as well as secondary bile acids, produced through microbial transformation of host-derived bile acids [17]. These metabolites interact with host receptors and signaling pathways, modulating lipid synthesis, oxidation, and transport. Thirdly, the gut microbiota influences fat deposition and lipid metabolism through complex inter-organ communication networks, notably the gut–liver axis and the gut–brain axis. The gut–liver axis facilitates bidirectional signaling via the portal circulation, allowing microbial metabolites and inflammatory factors to directly impact hepatic lipid metabolism [18]. The gut–brain axis integrates microbial signals with neuroendocrine pathways to regulate appetite, energy expenditure, and feeding behavior, thereby influencing overall energy balance and fat deposition [19].

Deciphering these host–microbiota interactions provides valuable insights into the biological drivers of production traits and offers promising opportunities for innovative nutritional interventions. Strategies targeting the gut microbiota, including probiotics, prebiotics, and synbiotics, are being actively explored to modulate fat deposition, improve feed efficiency, and enhance meat quality in livestock and poultry [20–22]. 

This review aims to summarize and discuss recent advancements in understanding the mechanisms by which the gut microbiota regulates fat deposition and lipid metabolism in production animals. We will focus on the roles of specific microbial taxa and their metabolites, with particular emphasis on the gut–liver and gut–brain axes, and highlight their implications for improving animal production efficiency and meat quality.

## Gut microorganisms associated with fat deposition

### Microbial composition in the intestine with different fat deposition levels

The gut microbiota, often referred to as the host’s “second genome” [20], plays a pivotal role in regulating lipid metabolism [21]. Compared to humans, many mammalian species harbor a more diverse and abundant intestinal microbial community, with microbial cells outnumbering host cells by nearly tenfold [22]. Based on comprehensive gene catalogs such as PIGC90, the gut microbiome can be classified into dozens of phyla, hundreds of genera, and thousands of species [23]. Among these, Firmicutes, Bacteroidetes, Proteobacteria, Spirochaetes, Tenericutes, and Actinobacteria are the most dominant phyla in the intestinal ecosystem. Although often viewed as a cohesive unit, the gut microbiota varies significantly across intestinal regions due to environmental factors such as pH, oxygen availability, and nutrient gradients [24]. Principal component analysis (PCA) has demonstrated that Firmicutes, Proteobacteria, and Bacteroidetes dominate the microbial communities of the duodenum, jejunum, and ileum. In contrast, the colon and cecum are primarily composed of Firmicutes and Bacteroidetes, which together account for over 90% of the microbial population [25].

Distinct differences in microbial composition have been observed across individuals with varying levels of fat accumulation, particularly in the jejunum and cecum—regions considered most relevant to adipogenesis [26, 27]. At the phylum level, individuals with higher fat content tend to exhibit increased abundances of Firmicutes, Proteobacteria, and Tenericutes, with Firmicutes being particularly enriched in the jejunum and colon [25, 26, 28]. Meanwhile, Proteobacteria and Tenericutes are more prevalent in the duodenum of high-fat individuals [25]. Conversely, lower-fat individuals typically show a higher relative abundance of Bacteroidetes, especially in the cecum and colon [28, 29]. However, phylum-level analysis can mask functional diversity, as microbial composition is also shaped by host genetics, diet, and analytical methods. Moreover, members within the same phylum can exhibit vastly different functional roles. Thus, genus and species level resolution is essential for a mechanistic understanding of how gut microbes influence fat deposition.

At the genus level, linear discriminant analysis effect size (LEfSe) has identified significantly higher levels of SCFA-producing genera—such as *Clostridium* and *Butyrivibrio*—in the colon of individuals with lower fat accumulation [25]. Metagenomic sequencing studies have shown that *Methanobrevibacter* is significantly more abundant in high-fat individuals (*P* < 0.05), whereas *Faecalibacterium prausnitzii* and *Ruminococcus* are enriched in leaner individuals [28]. Similarly, combined 16S rRNA and metagenomic analyses have revealed striking regional differences in microbial communities. For example, high-fat individuals exhibit increased abundances of *Clostridium phytofermentans*, *Lactobacillus johnsonii*, and *Escherichia fergusonii* in the jejunum; *Clostridium difficile*, *Clostridium sordellii*, and *Escherichia coli* in the ileum; and *Escherichia coli* and *Oscillibacter valericigenes* in the cecum. In contrast, lower-fat individuals display higher levels of *Paludibacter* and *Treponema succinifaciens* in the cecum, and increased abundances of *Actinobacillus succinogenes*, *Mannheimia succiniciproducens*, and *Cellulosilyticum lentocellum* in the jejunum and ileum [29]. Further integrative studies combining host genotyping and gut microbiota profiling have revealed associations between specific microbial taxa and host lipid phenotypes. For example, one study using a 50 K SNP genotyping array and 16S rRNA sequencing found that individuals with higher fat content had greater abundances of Prevotellaceae in the cecum, while leaner individuals had a predominance of *Spirochaeta* [26].

Together, these findings underscore the complex and region-specific relationships between gut microbial composition and host fat metabolism. A better understanding of these microbial signatures offers a theoretical basis for future interventions aimed at modulating lipid accumulation through targeted microbiota manipulation (Table 1).

**Table 1:** Distribution of intestinal microbiota associated with differential fat deposition levels

Taxa	Detection method	Fat deposition level	Intestinal segment	References
Firmicutes	-	High	Jejunum, colon	[25, 26, 28]
Proteobacteria	-	High	Duodenum	[25]
Tenericutes	-	High	Duodenum	[25]
Bacteroidetes	-	Low	Cecum, colon	[26, 27]
*Clostridium, Butyrivibrio*	LDA, LEfSe	Low	Colon	[25]
*Methanobrevibacter*	mNGS	High	Colon	[28]
*Faecalibacterium prausnitzii, Ruminococcus*	mNGS	Low	Colon	[28]
*Clostridium phytofermentans, Lactobacillus johnsonii*	16S rRNA	High	Jejunum	[29]
*Clostridium difficile, Clostridium sordellii, Escherichia coli*	16S rRNA	High	Ileum	[29]
*Escherichia coli, Oscillibacter valericigenes*	16S rRNA	High	Cecum	[29]
*Paludibacter, Treponema succinifaciens*	16S rRNA	Low	Cecum	[29]
*Actinobacillus succinogenes, Mannheimia succiniciproducens, Cellulosilyticum lentocellum*	16S rRNA	Low	Jejunum, ileum	[29]
Prevotellaceae	16S rRNA	High	Cecum	[26]
*Spirochaeta*	16S rRNA	Low	Cecum	[26]

“-” indicates unspecified detection method

*LDA* Linear discriminant analysis, *LEfSe* Linear discriminant analysis effect size, *mNGS* Metagenomic next-generation sequencing, *16S rRNA* High-throughput sequencing-based microbial profiling technology

### Gut microbiota composition and function in different adipose depots

Fat, as an essential nutrient and a key component of animals, directly influences meat production performance. In pigs, adipose tissue is mainly distributed in the subcutaneous fat, mesenteric fat, intestinal fat, intramuscular fat, intermuscular fat, and visceral fat. Among these, subcutaneous adipose tissue (SAT), visceral adipose tissue (VAT), and intramuscular fat (IMF) are of particular interest in animal husbandry due to their direct association with carcass composition and meat quality traits [30].

#### Subcutaneous adipose tissue (SAT)

Recent studies have revealed that various gut microbes are closely associated with subcutaneous fat deposition. Several beneficial microbes—such as *Clostridium butyricum*, *Stenotrophomonas* [26], *Limosilactobacillus reuteri*, *Bifidobacterium longum*, and *Eubacterium siraeum* play crucial roles in suppressing SAT accumulation. Specifically, *L. reuteri* can significantly reduce the mRNA expression of acetyl-CoA carboxylase 1 (*ACC1*), a rate-limiting enzyme in fatty acid synthesis, thereby inhibiting de novo lipogenesis in mice [31]. *B. longum* reduces SAT by promoting fatty acid oxidation [32] and enhancing duodenal barrier integrity [33]. Additionally, it modulates key genes involved in lipid metabolism, including AMP‐activated protein kinase (*AMPK*), peroxisome proliferator-activated receptor alpha (*PPARα*) and sterol regulatory element-binding protein 1 (*SREBP1*) to reduce VAT deposition [34]. *E. siraeum* lowers tyrosine levels in the gut and circulation through its metabolic activity, inhibiting the PI3K/AKT signaling pathway and subsequently reducing mTORC1 activity. This downregulates the expression of lipogenic genes such as *ACC* and fatty acid synthase (*FAS*), leading to decreased lipid accumulation in adipocytes. Moreover, *E. siraeum* and *C. butyricum* enhance lipid catabolism and energy expenditure by regulating genes in brown adipocytes and producing SCFAs, which help optimize the gut microbial composition [35].

Conversely, certain microbes are positively associated with SAT deposition, including *Prevotellaceae UCG-001*, *Alistipes*, *Clostridium *sensu stricto* 1* [26], and *Prevotella corpi*. *P. corpi* can stimulate the secretion of pro-inflammatory cytokines (TNF-α, IL-6, and IL-1β), activate the mTOR–HIF1α axis, upregulate lipogenic genes (e.g., *SCD1* and *FABP9*), and suppress lipolytic and energy metabolism genes such as *ADIPOR2* and *APOE*, ultimately promoting SAT accumulation [36].

#### Visceral adipose tissue (VAT)

While VAT serves as an important energy reservoir, its excessive accumulation can impair feed efficiency, inhibit muscle growth, and reduce lean meat yield and overall productivity [37]. Gut microbes positively correlated with VAT include *Clostridium butyricum*, *Faecalibacterium prausnitzii*, *Lactobacillus delbrueckii*, and *Methanobrevibacter* spp. *C. butyricum* activates the AMPK and peroxisome proliferator-activated receptor gamma (*PPARγ*) pathways via SCFA production [38], inhibits fatty acid synthase activity, and activates the Wnt10b signaling pathway to suppress adipose inflammation and hepatic lipid deposition [39]. *F. prausnitzii* secretes butyrate and other metabolites that improve insulin sensitivity and markedly reduce VAT accumulation in mice [40, 41]. *L. delbrueckii* promotes the conversion of cholesterol to bile acids, reduces hepatic lipid accumulation, downregulates hepatic *FAS* expression, increases butyrate levels, and activates AMPK signaling to suppress lipogenesis. It also upregulates the expression of lipoprotein lipase (LPL) and carnitine palmitoyltransferase 1 (CPT-1) in the liver and SAT, enhancing lipid breakdown and β-oxidation [42]. In contrast, *Methanobrevibacter* spp. reduce SCFA availability through methane production and upregulate lipid metabolism genes such as *ACC1* and *SREBP1*, thereby promoting VAT deposition [28].

#### Intramuscular fat (IMF)

IMF content is significantly and positively correlated with backfat thickness in pigs [43]. As a determinant of meat tenderness, leanness, and nutritional value, the regulation of IMF deposition has garnered increasing attention [44]. Evidence from farmed animals demonstrates that gut microbiota composition strongly influences IMF levels. For instance, *Prevotella stercorea* abundance was positively associated with IMF content and marbling scores in Jinhua pigs fed alfalfa-based diets [45]. Similarly, fecal microbiota transplantation (FMT) from Laiwu pigs with high IMF into Duroc pigs increased IMF deposition while reducing backfat thickness, highlighting a causal microbiota–IMF relationship [46]. In poultry, breast muscle IMF content ranged from 1.65% to 4.59%, and was strongly correlated with cecal microbiota-driven bile acid and riboflavin metabolism [47]. At the mechanistic level, *Bacillus* spp. can inhibit IMF accumulation by secreting antimicrobial peptides and SCFAs such as propionate, downregulating adipogenic transcription factors including *PPARγ* and *C/EBPα* [48]. The *Clostridium cluster XIVa* produces butyrate, activates the AMPK/PGC-1α pathway, enhances mitochondrial energy expenditure, and suppresses lipogenesis, thereby reducing IMF deposition [49]. Xie et al. [46] showed that *Bacteroides uniformis* and *Pyramidobacter piscolens* collaboratively inhibit AMPK activity, relieving its suppression of *SREBP1* and subsequently activating downstream lipogenic enzymes such as *FASN* and *ACACA*, thus forming a core regulatory network that promotes IMF accumulation. *Bifidobacterium pseudocatenulatum* promotes the biosynthesis of secondary bile acids, particularly lithocholic acid (LCA), activates the farnesoid X receptor (FXR), and thereby inhibits adipogenesis while promoting lipolysis to reduce SAT and optimize backfat thickness [50]. Notably, dietary glutamate supplementation significantly increases the relative abundance of Spirochaetota in the colon, which elevates colonic acetate levels and upregulates the expression of lipogenic genes (*ACC* and *FAS*), thus facilitating IMF deposition [51].

In summary, systematic studies across multiple gut segments and fat depots have progressively identified key microbial taxa and their mechanistic roles in regulating adipose deposition in pigs and poultry, providing a theoretical basis for precise microbial interventions in future pig production systems (Table 2).

**Table 2:** Classification of gut microbiota influencing porcine fat deposition

Adipose tissue type	Microbial taxa	Effect on fat deposition	Primary function	Model	Reference
Subcutaneous fat	*Clostridium butyricum*	Decrease	Enhances carbohydrate fermentation and improves feed efficiency	Pigs	[25]
	*Bifidobacterium longum*	Decrease	Promotes fatty acid oxidation	Mice	[32–34]
	*Stenotrophomonas* spp.	Decrease	–	Pigs	[26]
	*Eubacterium siraeum*	Decrease	Downregulates expression of lipogenic genes (*ACC* and *FAS*)	Pigs	[35]
	*Limosilactobacillus reuteri*	Decrease	Reduces mRNA level of *ACC1*	Mice	[31]
	*Bifidobacterium pseudocatenulatum*	Decrease	Enhances lithocholic acid (LCA) biosynthesis and activates FXR signaling pathway	Mice	[31]
	*Alistipes* spp.	Increase	–	Pigs	[26]
	*Prevotella* spp.	Increase	Induces inflammation, promotes lipogenesis, reduces lipolysis and energy expenditure	Pigs	[36]
Visceral fat	*Methanobrevibacter* spp.	Increase	Reduces energy utilization efficiency; upregulates *ACC1* and *SREBP1*	Pigs	[28]
	*Clostridium butyricum*	Decrease	Activates AMPK, *PPARγ*, Wnt10b pathways; inhibits *FAS* activity	Mice	[38, 39]
	*Faecalibacterium prausnitzii*	Decrease	Produces butyrate; improves insulin resistance	Mice	[40, 41]
	*Bifidobacterium longum*	Decrease	Regulates lipid metabolism-related genes such as AMPK, *PPARα*, and *SREBP1*	Mice	[33, 34]
	*Lactobacillus delbrueckii*	Decrease	Promotes bile acid excretion, inhibits lipogenesis, enhances lipid breakdown and oxidation	Pigs	[42]
Intramuscular fat	*Bacillus* spp.	Decrease	Downregulates adipogenic genes (e.g., *PPARγ*, *C/EBPα*)	Pigs	[48]
	*Clostridium cluster XIVa*	Decrease	Activates AMPK/PGC-1α pathway to increase mitochondrial energy expenditure	Pigs	[49]
	*Bacteroides uniformis*	Increase	Inhibits AMPK; activates downstream lipogenic enzymes such as *FASN* and *ACACA*	Pigs	[46]
	*Pyramidobacter piscolens*	Increase	Inhibits AMPK; activates downstream lipogenic enzymes such as *FASN* and *ACACA*	Pigs	[46]
	Spirochaetota	Increase	Increases colonic acetate production; upregulates lipogenic genes (*ACC* and *FAS*)	Pigs	[51]

### Gut microorganism for reducing fat deposition—*Clostridium butyricum*

Beyond depot- and species-specific observations, certain bacterial species have emerged as broadly useful models for microbial regulation of adiposity. In particular, *Clostridium butyricum* has attracted widespread attention because it consistently produces butyrate and other metabolites that modulate host energy metabolism, enhances intestinal barrier integrity and reduces endotoxemia, and reshapes the gut microbial community in ways that improve metabolic outcomes [52, 53]. Experimental work shows that oral administration or probiotic formulations of *C. butyricum* alleviate diet-induced obesity and associated metabolic dysfunction in mouse models [54], while separate studies in pigs and poultry report improvements in growth performance, gut health, and feed efficiency following *C. butyricum* supplementation [55]. These cross-species effects—observed in mice, piglets and poultry—justify treating *C. butyricum* as a representative, high-value species for mechanistic and translational research into microbiota-mediated control of fat deposition, and motivate the focused discussion that follows [56]. The following sections elaborate on the mechanisms by which *C. butyricum* mitigates fat deposition, from aspects of microbial reshaping, metabolite action, and gene regulation.

#### Microbial remodeling and cooperative proliferation

In high-fat diet-induced obese mouse models, a marked increase in Firmicutes and a reduction in Bacteroidetes have been observed, indicating gut dysbiosis. Supplementation with *C. butyricum* not only increased its own abundance in the host gut but also helped rebalance the Firmicutes/Bacteroidetes ratio [57]. This microbial restructuring may improve overall metabolic function and contribute to reduced fat accumulation. Additionally, *C. butyricum* facilitates the proliferation of other beneficial bacteria such as *Bifidobacterium* and *Akkermansia muciniphila* [21]. These bacteria influence lipid metabolism by producing SCFAs, regulating hepatic genes involved in fatty acid metabolism, and enhancing intestinal barrier integrity, ultimately reducing lipid storage in the body [58].

Liao et al. [52] employed 16S rDNA amplicon sequencing on fecal samples and found that high-fat diet increased the abundance of Firmicutes and Actinobacteria. Intervention with *C. butyricum* strains such as C20, Z1T1, 47T7, and L1T1 significantly reduced the proportion of Actinobacteria. Specifically, strains C20 and Z1T1 inhibited the growth of genera like *Mesorhizobium*, *Coriobacteriaceae UCG-002*, and *Erysipelatoclostridium*, while increasing the abundance of Muribaculaceae, thereby improving gut microbial ecology and indirectly influencing lipid metabolism and deposition.

#### Metabolite activity and signaling pathway regulation

The most prominent characteristic of *C. butyricum* is its strong ability to produce butyrate. As a key SCFA, butyrate not only serves as an energy source for colonocytes but also plays a critical role in host energy metabolism and signaling, significantly enhancing total SCFA levels and reducing fat accumulation [57]. Studies on Jinhua pigs revealed that colonic butyrate concentrations were significantly higher in low-fat groups, with elevated expression of key butyrate-producing genes, phosphate butyryltransferase (*ptb*) and butyrate kinase (*buk*), suggesting a pivotal role for butyrate in suppressing lipid storage and obesity development [25].

In vitro experiments further revealed the molecular mechanism by which *C. butyricum* modulates lipid metabolism. Zhao et al. [58] co-cultured various concentrations of *C. butyricum* with Caco-2 cells and found that the bacterium significantly upregulated the mRNA and protein expression of angiopoietin-like 4 (ANGPTL4), without impairing cell viability. ANGPTL4, an inhibitor of LPL, reduces the uptake and deposition of circulating lipids into adipocytes, thereby playing an essential role in lipid homeostasis [59]. Untargeted metabolomics studies further demonstrated that *C. butyricum* substantially alters tryptophan and purine metabolic profiles in the gut. Using LC-MS to analyze fecal metabolites, Liao et al. [52] observed increased levels of indole derivatives—tryptophan metabolites—that can suppress miR-181 expression in white adipose tissue and promote thermogenesis, thus combating obesity. Meanwhile, purine metabolites such as adenosine and inosine were found to enhance fat tissue thermogenic capacity and modulate immune responses to reduce fat deposition. These metabolic changes were significantly associated with decreased host fat content.

Pathway enrichment analysis indicated that *C. butyricum* may reduce fat accumulation by regulating carbohydrate-active enzymes (CAZymes). In obese mice, *C. butyricum* treatment notably increased the abundance of the CAZyme family GH13, which is critical for starch degradation. Enhanced degradation of insoluble starch by GH13 reduces the substrates available for lipogenesis, thus potentially lowering fat deposition [25]. Moreover, accumulating evidence shows that *C. butyricum* and its metabolites can directly inhibit fat synthesis and promote lipolysis by downregulating genes related to lipid synthesis (e.g., *FAS* and *ACC1*) and upregulating those involved in β-oxidation and energy expenditure (e.g., *CPT1* and *UCP* family), thereby effectively reducing lipid accumulation [39].

## Mechanistic analysis of the gut microbiota–gut–liver axis on fat metabolism

### Basic mechanisms of the gut–liver axis and disruption of microbiota–gut–liver axis homeostasis

The gut–liver axis refers to the bidirectional regulatory network formed between the gut and liver through nutrient absorption, microbial metabolite release, and immune signaling, mediated primarily by the portal vein circulation and bile acid circulation [60]. Under normal physiological conditions, the gut microbiota ferments dietary fibers to produce SCFAs (such as butyrate and propionate) and converts primary bile acids. These metabolites enter the liver via the portal vein, where they participate in regulating hepatic lipid synthesis, oxidation, and energy metabolism. Conversely, the liver synthesizes and secretes primary bile acids into the intestine, where gut microbes (e.g., *Clostridium* species) convert them into secondary bile acids (e.g., deoxycholic acid, DCA). These secondary bile acids then act on hepatic or intestinal nuclear receptors such as the farnesoid X receptor (FXR) and membrane receptors like Takeda G protein-coupled bile acid receptor 5 (TGR5), which further inhibit SREBP-1c-mediated lipogenesis and promote lipid catabolism in the liver [61, 62]. Additionally, SCFAs can activate hepatic G-protein coupled receptors (GPR41/43) to directly suppress hepatic de novo lipogenesis (DNL) and promote mitochondrial β-oxidation [63]. SCFAs also inhibit histone deacetylase (HDAC) activity, facilitating liver tissue regeneration and repair in mice [64]. Given the conserved nature of SCFA signaling, these mechanisms may likewise support improved hepatic lipid handling and meat quality in pigs. Collectively, this network maintains lipid metabolic homeostasis in the host, and disruption of this axis can lead to metabolic disorders such as fatty liver disease and insulin resistance [65].

Disruption of gut–liver axis homeostasis is primarily caused by impaired intestinal barrier function and dysbiosis of the gut microbiota. The intestinal barrier integrity is essential for maintaining gut–liver axis stability. This barrier comprises mechanical components (tight junction proteins such as ZO-1 and occludin), chemical barriers (mucus layer), immune defenses (IgA and antimicrobial peptides), and microbial barriers [66]. Once the barrier is compromised, endotoxins such as lipopolysaccharides (LPS) and pathogen-associated molecular patterns (PAMPs) can translocate through the epithelial gap into the portal circulation and reach the liver, activating Toll-like receptor 4 (TLR4) and downstream NF-κB inflammatory signaling pathways, triggering hepatic insulin resistance and steatosis [67, 68]. LPS is a major component of the outer membrane of Gram-negative bacteria [69]. Specifically, LPS binds to TLR4 and co-receptors CD14/MD-2 on intestinal epithelial cells and hepatic Kupffer cells, inducing endocytosis and initiating MyD88-dependent signaling cascades. This activates TRAF6, TAK1, and the IKK complex, amplifying NF-κB and MAPK pathways, and leading to the release of pro-inflammatory cytokines (TNF-α, IL-6, and IL-1β) and chemokines [70, 71]. Persistent inflammation not only suppresses hepatic bile acid synthesis and secretion, disturbing the gut–liver circulation, but also induces abnormal phosphorylation of insulin receptor substrate 1 (IRS-1 at Ser616) via inflammatory cytokines, impairing insulin signaling and exacerbating hepatic lipid metabolism dysregulation [72].

In porcine models, Proteobacteria such as *Escherichia coli* and Bacteroidetes species like *Prevotella copri* have been identified as major sources of LPS [36, 73]. Dysbiosis marked by an increased proportion of Gram-negative bacteria leads to elevated intestinal LPS levels [74]. High-fat diets or alcohol exposure reduce expression of tight junction proteins, aggravating LPS leakage and portal translocation, thereby activating hepatic stellate cells (HSCs) and promoting fibrosis progression [75]. Furthermore, intestinal PPARα signaling maintains gut vascular barrier integrity by upregulating TRIM38 expression; loss of PPARα function causes endotoxemia and hepatic immune imbalance, worsening fatty liver disease [76].

There is a bidirectional feedback loop between intestinal inflammation and bile acid metabolism dysregulation: hepatic inflammation triggered by LPS suppresses bile acid synthesis and secretion, while intestinal inflammation compromises barrier integrity and alters microbiota-mediated bile acid conversion, resulting in abnormal proportions of secondary bile acids. This bile acid dysregulation further disturbs gut microbiota homeostasis, increasing LPS permeability and inflammatory responses [77]. Notably, elevated LPS levels in pigs strongly correlate with obesity phenotypes, mainly by inducing insulin resistance that indirectly affects lipid metabolism [78]. However, the direct molecular mechanisms by which LPS regulates lipid metabolism remain to be elucidated.

**Fig. 1:**
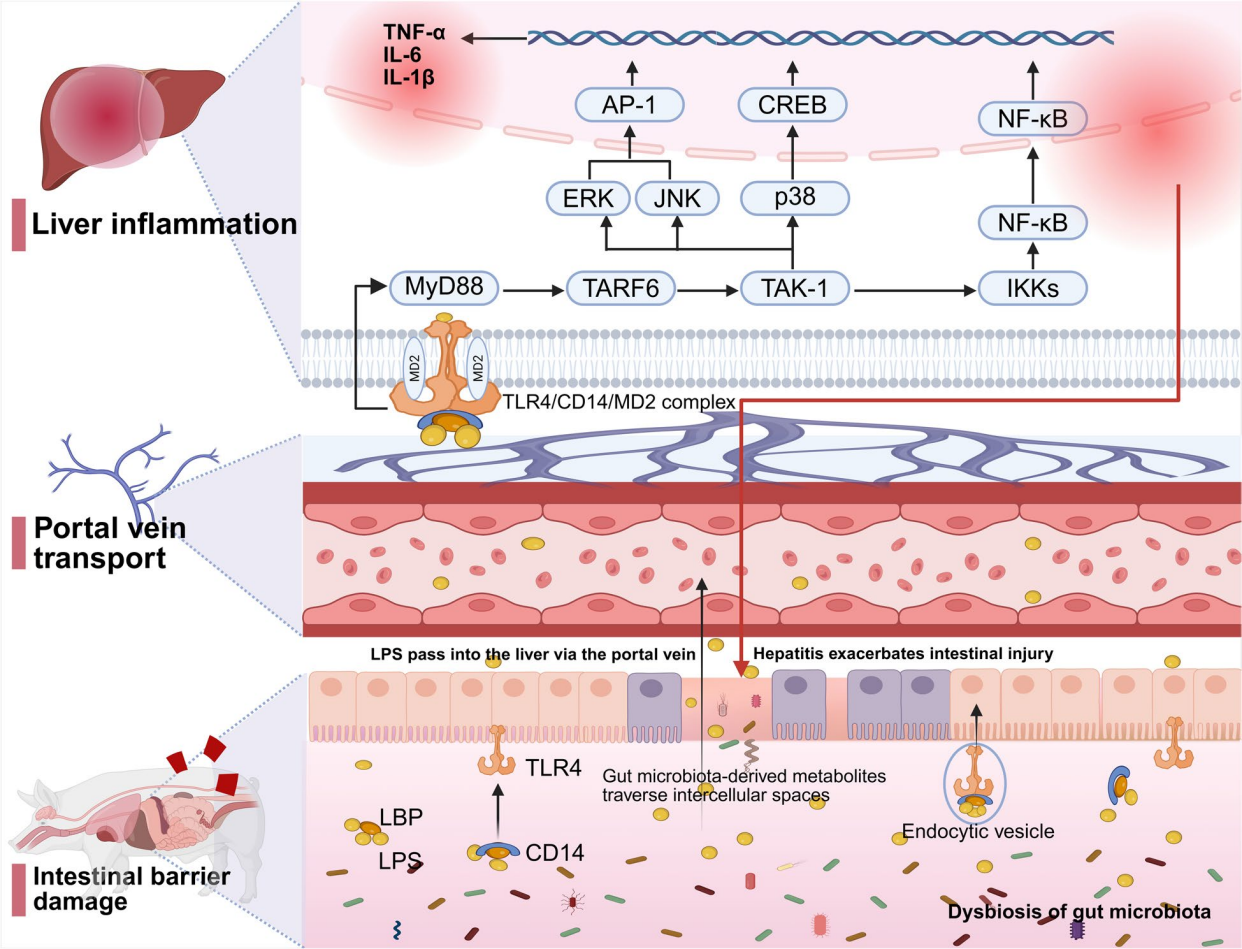
The underlying mechanisms of disrupted homeostasis in the microbiota–gut–liver axis. TLR4, Toll-like receptor 4; MyD88, myeloid differentiation primary response protein 88; TRAF6, TNF receptor-associated factor 6; TAK-1, TGF-beta-activated kinase 1; ERK, extracellular signal-regulated kinase; JNK, c-Jun N-terminal kinase; p38, p38 mitogen-activated protein kinase; NF-κB, nuclear factor kappa-B; IKKs, IκB kinases; AP-1, activator protein 1; CREB, cAMP response element-binding protein; TNF-α, tumor necrosis factor alpha; IL, interleukin; MD2, myeloid differentiation factor 2; CD14, cluster of differentiation 14; LBP, lipopolysaccharide-binding protein; LPS, lipopolysaccharide

In summary, disruption of the gut–liver axis homeostasis is a key pathological basis for abnormal lipid metabolism and excessive fat deposition. Future studies should focus on the molecular networks linking gut microbiota and gut–liver axis imbalance, aiming to develop targeted interventions for this axis as novel therapeutic strategies (Fig. 1).

### Regulation of the gut–liver axis and fat deposition by microbiota derived SCFAs

Short-chain fatty acids, a group of saturated fatty acids with 1–6 carbon atoms, mainly include acetate, propionate, and butyrate. These SCFAs are primarily synthesized through anaerobic fermentation of indigestible carbohydrates such as dietary fiber and resistant starch by gut microbiota [79, 80]. Among them, acetate is the most abundant SCFA (accounting for 60%–70%) and is mainly produced by *Akkermansia muciniphila* [81] and *Desulfovibrio vulgaris* [82] via the pyruvate dehydrogenase complex (PDC), which converts pyruvate to acetyl-CoA, followed by acetyl-CoA synthetase (ACS)-mediated synthesis of acetate. Additionally, some acetate-producing bacteria can directly synthesize acetate from CO_2_ and H_2_ through the Wood–Ljungdahl pathway [83].

Propionate (approximately 20%) is synthesized via three major pathways: (1) the succinate pathway, where dominant Bacteroidetes convert succinate to methylmalonyl-CoA, then to propionate; (2) the acrylate pathway, where lactate is used as a precursor and converted to propionate through the intermediate acryloyl-CoA; and (3) the propanediol pathway, where fermentation of deoxy sugars (e.g., fucose and rhamnose) generates 1,2-propanediol, which is further converted into propionate via acryloyl-CoA [84]. Representative propionate-producing bacteria include *Veillonella* [85], *Akkermansia muciniphila* [86, 87], and *Limosilactobacillus reuteri* [88].

Butyrate (about 10%–20%) is primarily produced by members of the phylum Firmicutes, such as Lachnospiraceae [89, 90], *Ruminococcus* [91], *Clostridium butyricum* [25], *Faecalibacterium prausnitzii*, and *Eubacterium rectale* [92]. The core synthesis pathways include: (1) the butyrate kinase pathway, in which two molecules of acetyl-CoA condense to form butyryl-CoA, which is then phosphorylated by butyrate kinase to produce butyrate; and (2) the butyryl-CoA/acetyl-CoA transferase pathway, in which butyrate is generated through CoA exchange between butyryl-CoA and acetyl-CoA [93].

Most SCFAs are rapidly absorbed by colonic epithelial cells, with the remainder entering the liver via the portal vein. In the colon, SCFAs predominantly exist in their anionic form (SCFA^−^) and require specific transporters for cellular uptake. These include monocarboxylate transporter 1 (MCT1, encoded by *SLC16A1*) and sodium‐coupled monocarboxylate transporter 1 (SMCT1, encoded by *SLC5A8*) [94]. MCT1 is localized on both the apical and basolateral membranes of colonocytes, where it symports SCFA^−^ together with H^+^ into the cell. In contrast, SMCT1 is primarily expressed on the apical membrane and co‐transports SCFA^−^ with two Na^+^ ions, utilizing the sodium gradient to drive uptake [95]. SCFAs not metabolized by colonocytes enter the mesenteric veins (both superior and inferior) and subsequently drain into the portal vein [96]; hepatocyte basolateral membranes express MCT1 and MCT4, which mediate further transmembrane uptake of SCFAs (in pigs) [97, 98].

Within the colon, butyrate serves as the primary energy substrate for epithelial cells. Butyrate inhibits HDAC activity and upregulates the aryl hydrocarbon receptor (AhR) and hypoxia‐inducible factor 1α (HIF1α), thereby promoting interleukin‐22 (IL-22) production, maintaining mucosal barrier integrity, and preserving immune homeostasis to suppress intestinal inflammation [99, 100]. SCFAs also attenuate gut inflammation via activation of G protein-coupled receptor (GPCR) pathways. Specifically, SCFAs upregulate expression of GPR41 and GPR43, and binding to GPR43 directs neutrophil chemotaxis toward sites of inflammation. Moreover, GPR109A activation by butyrate induces differentiation of regulatory T cells (Tregs) and IL-10-secreting T cells, thereby inhibiting colitis [101, 102]. By engaging GPR41 and GPR43 on intestinal and immune cells, SCFAs further suppress proinflammatory JAK2/STAT3 signaling cascades, effectively reducing inflammatory responses [103].

The integrity of the intestinal barrier is closely linked to the state of gut inflammation. In experimental models wherein sulforaphane supplementation reshapes the gut microbiota, SCFAs have been shown to upregulate tight junction proteins (e.g., ZO-1 and occludin), thereby reducing intestinal permeability, lowering circulating endotoxin (LPS) levels, and markedly alleviating colonic inflammation [104]. Concurrently, butyrate stimulates goblet cells to secrete mucus proteins (such as MUC2), which form a protective mucous layer that impedes pathogen invasion and preserves barrier integrity [105]. Moreover, SCFAs can modulate luminal pH, thereby altering the composition of the porcine gut microbiota and further mitigating intestinal inflammation.

Upon entry into the liver, propionate constitutes the predominant SCFA. In vivo studies have demonstrated that propionate supplementation reduces plasma low‐density lipoprotein (LDL) and total cholesterol levels [106]. One proposed mechanism involves intestinal immune modulation leading to downregulation of the cholesterol transporter NPC1L1, which diminishes enteric cholesterol uptake and, in turn, reduces hepatic cholesterol influx and very low-density lipoprotein (VLDL) secretion [107]. Butyrate also exerts epigenetic effects to lower hepatic cholesterol. In a deoxynivalenol (DON)‐induced piglet liver injury model, sodium butyrate (NaBu) inhibits histone H3K27 and H3K9 acetylation, thereby reducing transcriptional activity of key cholesterol biosynthesis genes (*HMGCR*, *SQLE*, and *DHCR24*). Concurrently, butyrate downregulates the nuclear receptor RORγ, preventing its binding to promoters of cholesterol synthesis genes, thereby collectively suppressing the biosynthetic pathway [108]. Furthermore, in rodent models of nonalcoholic fatty liver disease (NAFLD), SCFAs have been found to inhibit NF-κB activation in hepatocytes, ameliorating hepatic inflammation [109].

Gut microbiota-derived SCFAs can regulate adipose deposition by simultaneously inhibiting lipogenesis and promoting fatty acid oxidation. SCFAs primarily enhance glucagon‐like peptide-1 (GLP-1) secretion through activation of G protein-coupled receptors (GPCRs) on intestinal L cells [110]. For instance, Han et al. [111] used fecal microbiota transplantation–ileal anastomosis (FMT-KNGT) to modulate the murine gut microbiome, reporting that enrichment of SCFA-producing bacteria led to significant increases in acetate and butyrate levels, which in turn robustly activated GPR43 and boosted GLP-1 release. Similarly, oral administration of a polyherbal concoction containing *Pueraria*, *Morus alba*, and *Morus rubra* enhanced cecal hexanoate (an SCFA) concentrations in mice; SCFAs bound to GPR43, elevating serum GLP-1 levels [112]. Parallel feeding studies corroborate that elevated SCFA production and GPR41 activation are also tightly associated with enhanced GLP-1 secretion [113, 114]. Moreover, SCFAs may stimulate GLP-1 release by activating free fatty acid receptors FFAR2/FFAR3 on enteroendocrine cells and/or by engaging ERK and p38 MAPK signaling pathways [115, 116].

**Fig. 2:**
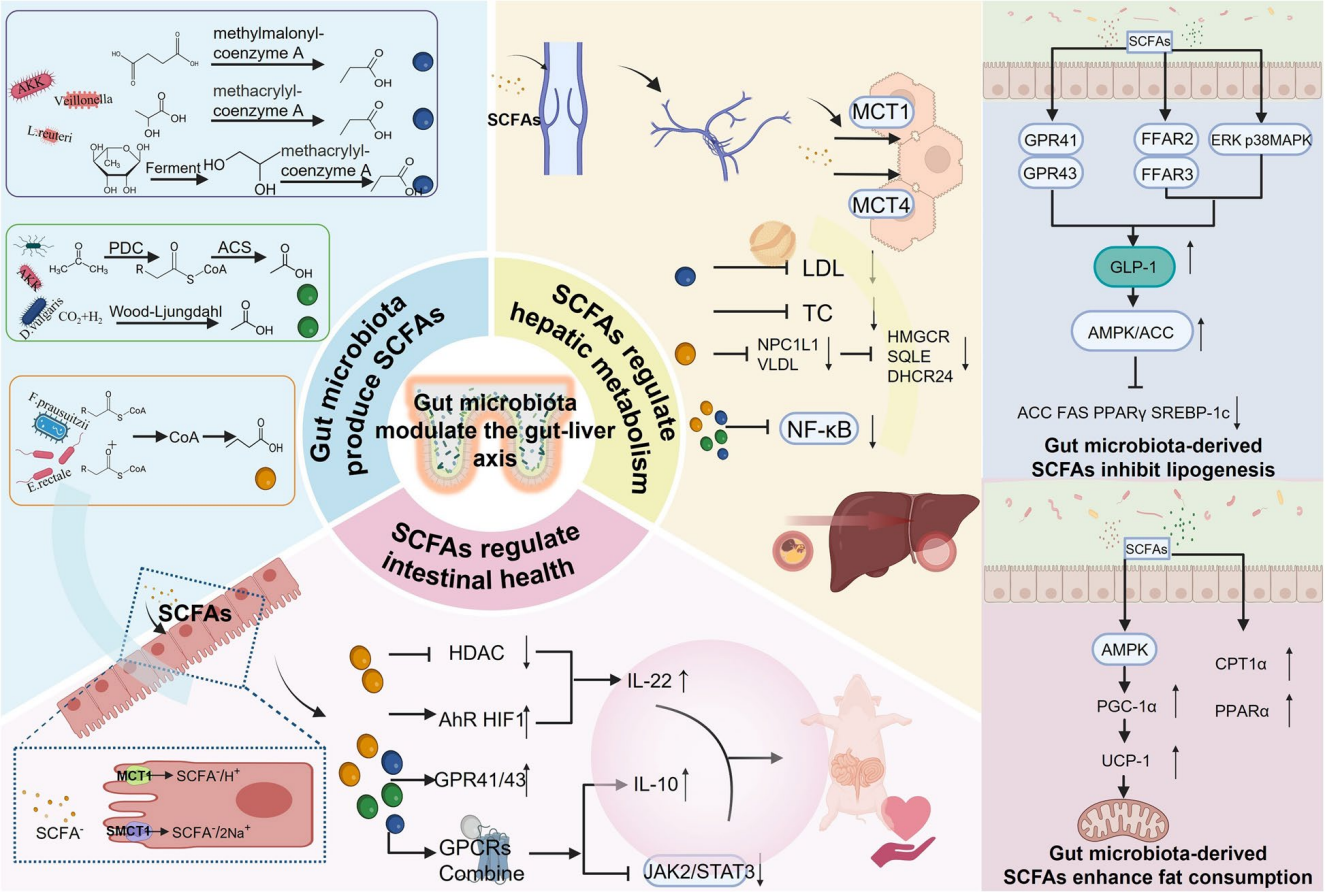
The underlying mechanisms of intestinal microbiota regulation of the gut–liver axis in modulating fat deposition. Once absorbed by the intestine, microbiota‐derived SCFAs attenuate hepatic inflammation and remodel the gut microbial community, thereby reducing intestinal permeability. These SCFAs regulate fat deposition primarily by inhibiting de novo lipogenesis and enhancing lipid oxidation. SCFAs, short-chain fatty acids; LDL, low-density lipoprotein; TC, total cholesterol; VLDL, very-low-density lipoprotein; MCT, monocarboxylate transporter; SMCT1, sodium-coupled monocarboxylate transporter 1; HMGCR, HMG-CoA reductase; SQLE, squalene epoxidase; DHCR24, 24-dehydrocholesterol reductase; ACC, acetyl-CoA carboxylase; FAS, fatty acid synthase; PPARγ, peroxisome proliferator-activated receptor gamma; SREBP-1c, sterol regulatory element-binding protein 1c; AMPK, AMP-activated protein kinase; CPT1α, carnitine palmitoyltransferase 1α; PGC-1α, PPARγ coactivator 1-alpha; UCP-1, uncoupling protein 1; GPR, G-protein coupled receptor; FFAR, free fatty acid receptor; GPCRs, G-protein coupled receptors; AhR, aryl hydrocarbon receptor; HIF, hypoxia-inducible factor; NF-κB, nuclear factor kappa-B; IL, interleukin; JAK2/STAT3, Janus kinase 2/signal transducer and activator of transcription 3; GLP-1, glucagon-like peptide 1

GLP-1 and its analogs activate the AMPK/ACC phosphorylation cascade, downregulating expression of lipogenic genes (*ACC*, *FAS*, and *PPARγ*) and inhibiting hepatic *SREBP-1c*, thereby reducing triglyceride accumulation and suppressing de novo lipogenesis [117]. SCFAs can also directly activate AMPK, increasing levels of phosphorylated AMPK (p-AMPK), which upregulates peroxisome proliferator-activated receptor γ coactivator 1α (*PGC-1α*). *PGC-1α* subsequently drives uncoupling protein-1 (UCP-1) expression, augmenting mitochondrial function and promoting fatty acid oxidation [118]. For example, *Desulfovibrio vulgaris* treatment in mice significantly elevated systemic acetate levels and concomitantly upregulated carnitine palmitoyltransferase 1a (CPT1a) and *PPARα* expression, indicating that SCFAs enhance mitochondrial β-oxidation capacity and reduce hepatic lipid deposition [82] (Fig. 2).

### Bile acids in the microbiota–gut–liver axis regulating fat deposition

Bile acids (BAs) are amphipathic steroidal compounds that occupy a central role in microbiota–gut–liver axis signaling [119]. Depending on their biosynthetic origin, bile acids are classified as primary or secondary. The liver is the principal site for primary bile acid synthesis, where cholesterol is converted via the classical (CYP7A1‐mediated) and alternative (CYP27A1‐mediated) pathways into cholic acid (CA) and chenodeoxycholic acid (CDCA), respectively [120]. In swine, primary bile acids also include hyocholic acid (HCA). The gut microbiota does not perform de novo bile acid synthesis; instead, primary bile acids secreted into the bile duct enter the intestinal lumen and are converted by commensal bacteria into secondary bile acids (SBAs) such as deoxycholic acid (DCA) and LCA. This biotransformation is initiated by bacterial bile salt hydrolases (BSHs), which deconjugate CA into DCA and CDCA into LCA [121]. In pigs, the gut microbiota additionally contributes to the formation of hyodeoxycholic acid (HDCA) [122]. Although research on porcine intestinal BSHs remains incomplete, BSH activity is highly conserved across all major gut bacterial phyla (Bacteroidetes, Clostridiales, Actinobacteria) [123]. Subsequently, select gut bacteria—such as *Clostridium scindens* and *Eubacterium* spp.—express bile acid‐inducible (*bai*) genes that catalyze the 7α‐ or 7β‐dehydroxylation removal from primary bile acids, yielding SBAs [124]. The microbiota further stereoisomerizes bile acids: *Clostridium scindens*, *Clostridium hylemonae*, and *Peptacetobacter hiranonis* utilize hydroxysteroid dehydrogenases (HSDHs) to oxidize hydroxyl groups to keto‐groups, whereas *Ruminococcus gnavus*, *Clostridium baratii*, and *Collinsella aerofaciens* are capable of epimerizing hydroxyl moieties (e.g., converting CDCA into the anti‐inflammatory ursodeoxycholic acid [UDCA]). In addition, *Eggerthella lenta* and related strains can modify multiple hydroxyl positions to generate diverse stereoisomers, thereby exerting profound regulation over host metabolism and inflammatory responses [125].

Conjugated bile acids synthesized in hepatocytes are secreted into bile canaliculi via bile salt export pumps (BSEP; encoded by *ABCB11*) and other transporters, stored in the gallbladder, and released into the duodenum upon feeding‐induced gallbladder contraction [126]. In the small intestine, bile acids emulsify dietary lipids and facilitate the absorption of fats and fat‐soluble vitamins. The majority of bile acids (including SBAs) are reabsorbed in the terminal ileum through the apical sodium-dependent bile acid transporter (ASBT) [127]. Reabsorbed bile acids exit enterocytes via the organic solute transporter α/β (OSTα/β) into the portal circulation [99] and return to the liver via the portal vein. Hepatocyte basolateral membranes express the sodium-taurocholate cotransporting polypeptide (NTCP) and organic anion transporting polypeptides (OATPs), which mediate efficient hepatic uptake. Once inside hepatocytes, free bile acids are reconjugated, and the newly formed conjugated bile acids, together with de novo-synthesized conjugated bile acids, are secreted again into bile, thereby completing the enterohepatic circulation [128, 129].

The composition and metabolic activity of the gut microbiota critically determine the species and relative proportions of SBAs, which in turn modulate downstream signaling pathways. Conversely, bile acids reciprocally shape the microbial community structure. For example, the porcine‐specific SBA hyodeoxycholic acid selectively enriches *Parabacteroides distasonis* populations in the gut [130]. Exogenous bile acid supplementation also alters the microbiota: dietary inclusion of CDCA in weaned piglets increases the relative abundance of *Prevotella 9* and *Prevotellaceae TCG‐001* while reducing that of *Dorea* [131]. Spearman correlation analysis further reveals that LCA positively correlates with the abundance of *Bifidobacterium pseudolongum* in the porcine colon [50].

Bile acids regulate porcine lipid metabolism and adiposity primarily by activating two nuclear and membrane receptors: FXR and TGR5. FXR is highly expressed in the liver and ileum; upon activation by bile acids, FXR induces expression of the small heterodimer partner (*SHP*), which suppresses transcription of *CYP7A1*, the rate-limiting enzyme in bile acid synthesis, thus establishing a negative feedback loop to maintain bile acid homeostasis [132]. The FXR/SHP axis also downregulates the lipogenic transcription factor *SREBP-1c* and upregulates the fatty acid oxidation regulator *PPARα*, thereby inhibiting hepatic de novo lipogenesis and triglyceride accumulation [133]. TGR5 is widely expressed in intestinal L cells, macrophages, skeletal muscle, and brown adipose tissue. Bile acid-mediated TGR5 activation elevates intracellular cyclic AMP levels, promoting glucagon‐like peptide-1 (GLP-1) secretion and improving glucose homeostasis [134]. In brown adipose tissue and skeletal muscle, TGR5 activation via sympathetic nervous system signaling enhances expression of hormone‐sensitive lipase (HSL) and UCP-1, thereby increasing lipolysis and thermogenesis [135].

Critically, FXR and TGR5 are not simply redundant: they can act complementarily or antagonistically depending on bile-acid species, concentration, tissue distribution and host metabolic state [136]. Importantly, these “concentration-dependent effects” can be partly quantified. For instance, CDCA is a potent endogenous agonist of FXR with an EC50 in the low micromolar range, whereas LCA and DCA are among the strongest natural agonists for TGR5, with EC50 values typically below 1 μmol/L [137]. In vivo, physiological bile acid concentrations in the porcine intestine and portal circulation are usually maintained in the 10–100 μmol/L range, while pharmacological supplementation can elevate them to supraphysiological levels [138]. This difference explains why some studies report lipolytic or anti-adipogenic effects (via balanced FXR–TGR5 signaling), whereas others observe pro-adipogenic outcomes under biased or excessive FXR activation. When both receptors are appropriately engaged (for example by a balanced pool of secondary bile acids that provide ligands for FXR and G-protein signaling for TGR5), FXR-driven suppression of lipogenesis combined with TGR5-mediated enhancement of energy expenditure yields coordinated reductions in fat deposition [139]. Conversely, excessive or biased FXR activation can reduce the hepatic synthesis or intestinal availability of TGR5 agonists (i.e., alter the bile-acid pool), thereby blunting TGR5’s lipolytic/thermogenic contributions and producing net neutral or even pro-adipogenic outcomes [140]. For porcine lipid biology, these interactions suggest two practical implications. First, different bile-acid species (and their conjugation/isomer forms) will variably engage FXR vs. TGR5 and thus produce distinct lipid phenotypes; reporting the bile-acid profile (including LCA/DCA/HDCA and conjugates) is therefore essential [141]. Second, interventions aimed at modifying bile acids (microbiota modulation, dietary BA supplements) should be designed to preserve or recreate a balanced FXR–TGR5 signaling state rather than strongly favoring one receptor.

Moreover, different bile acid species exert distinct regulatory effects on lipid metabolism. HDCA suppresses gene expression of *ACC* and *PPARγ*, both key regulators of lipogenesis and adipocyte differentiation, while upregulating HSL to promote lipolysis, thus significantly reducing fat accumulation [122]. In a Bacteroides ovatus-colonized murine model, *Clostridium scindens* elevates intestinal HDCA levels; HDCA, in turn, enhances TGR5 expression and suppresses FXR expression, leading to increased GLP-1 secretion [142]. Although results from the murine model suggest that HDCA may act via the TGR5/FXR axis, whether this mechanism is fully conserved in swine warrants further direct validation, given the unique characteristics of porcine bile acid metabolism. Conversely, LCA appears to promote adiposity: it upregulates PPARγ at both the mRNA and protein levels and activates lipogenic genes *SREBP-1c* and *FAS*, thereby augmenting lipid synthesis and resulting in increased backfat thickness [122]. However, the role of LCA in porcine lipid metabolism remains contentious. On the one hand, supraphysiological supplementation of LCA has been shown to favor lipogenesis, as described above; on the other hand, evidence from both pigs and rodents indicates that low-dose LCA (0.3%) supplementation or microbiota-driven SBA synthesis can reduce fat deposition by selectively engaging TGR5 signaling [50]. Supporting this mechanism, LCA is recognized as one of the most potent natural TGR5 agonists, and TGR5 activation has been demonstrated to increase GLP-1 secretion, enhance thermogenesis, and protect against diet-induced obesity (in mice) [135, 143]. Collectively, these findings highlight that the metabolic role of LCA is highly dose- and context-dependent: while excessive or FXR-biased activation may promote lipid accumulation, physiological concentrations acting through TGR5 can confer protective, anti-adipogenic effects. Clarifying these variables in porcine models will be essential for resolving current discrepancies and for designing precise dietary or microbial interventions targeting bile acid metabolism in swine. Notably, Zha’s study, which more closely mimics physiological conditions by leveraging “microbiota-host co‐metabolism” [50], provides translational evidence for LCA’s protective metabolic role in an obesity model. In summary, current evidence underscores that LCA exerts dual and context-dependent roles in porcine lipid metabolism. While supraphysiological exposure enhances lipogenesis and backfat deposition, low-dose or microbiota-derived LCA has been shown in pigs to attenuate adiposity, largely via TGR5-mediated pathways. These findings highlight the importance of carefully distinguishing between experimental conditions, and emphasize that porcine-specific data provide the most relevant evidence for resolving this controversy.

Elucidating these variables’ effects on experimental outcomes will inform the development of more precise porcine models and optimize dosing and intervention strategies in future studies.

### Microbiota–gut–adipose axis: intestinal morphological and endocrine mechanisms connecting gut microbiota to adipose tissue

In pigs, morphological differences in the gastrointestinal tract appear to modulate fat deposition by influencing nutrient absorption and barrier function. For example, Huainan pigs, which are characterised by higher intramuscular fat content, exhibit deeper intestinal crypts and a higher number of goblet cells in the rectum and colon than lean Large White pigs. These structural differences indicate enhanced epithelial integrity and nutrient absorption capacity. Microbial analyses revealed that Huainan pigs have a higher Firmicutes/Bacteroidetes ratio and are dominated by *Lactobacillus* species in distal gut segments. These bacteria are known to produce SCFAs and conjugated secondary bile acids, which strengthen the epithelial barrier and facilitate micelle formation for fatty acid uptake, thereby promoting lipid absorption and intramuscular fat deposition [144]. Integrated microbiota-metabolite analyses further showed that *Lactobacillus* correlates with conjugated secondary bile acids, forming a host–microbe–metabolite network that enhances bile acid recycling and lipid absorption while maintaining an anti-inflammatory intestinal environment [13].

Interactions between adipose tissue, the liver and the gut also illustrate the gut’s role as a communication hub. In a porcine model in which uncoupling protein 1 (UCP1) is knocked into white adipose tissue, WAT-derived UCP1 suppressed the expression of cholesterol-homeostasis and bile acid biosynthesis genes in both inguinal white adipose tissue and the liver. This reduction in bile acid synthesis altered the pool of bile acids entering the intestinal lumen and reshaped the gut microbiota, suggesting an adipose–liver–gut axis whereby adipose tissue affects gut microbial composition via the liver [145].

Conversely, gut-derived signals can modulate peripheral lipid metabolism. Obese Jinhua pigs show higher expression of hepatic lipogenic enzymes and lower intestinal expression of ANGPTL4, an inhibitor of LPL, compared with lean Landrace pigs. Faecal microbiota transplantation from Jinhua pigs into germ-free mice decreases intestinal ANGPTL4 expression and increases host adiposity, indicating that gut microbiota regulate intestinal ANGPTL4 and thereby control peripheral triglyceride clearance and fat deposition [146]. These findings underscore the intestine’s endocrine function in transmitting microbial signals to adipose tissue via hormones and lipid transport regulators.

In poultry, the gut–adipose connection has also been documented. Chickens with high abdominal fat deposition exhibit increased hepatic expression of lipogenic genes (*ACSL1*, *FADS1*, *CYP2C45*, *ACC*, and *FAS*) and reduced expression of fat catabolism genes (*CPT-1* and *PPARα*) and lipid transport gene *APOAI*. These obese birds harbour a cecal microbiota enriched in *Parabacteroides*, *Parasutterella*, *Oscillibacter* and *Anaerofustis*, whereas lean birds have more Sphaerochaeta. Transfer of microbiota from lean donors reduces the expression of lipogenesis genes, increases fat catabolism genes and decreases abdominal fat deposition. Specific microbial taxa can either promote or inhibit fat deposition: *Enterococcus faecium* and *Klebsiella/Escherichia-Shigella* enhance lipogenesis and hyperlipidaemia, whereas *Lactobacillus johnsonii* and *Microbacterium/Sphingomonas* stimulate LPL activity and fat catabolism [147]. These examples highlight how intestinal microbial communities in poultry communicate with the liver and adipose tissue through metabolites and bile acids to regulate fat accumulation.

**Fig. 3:**
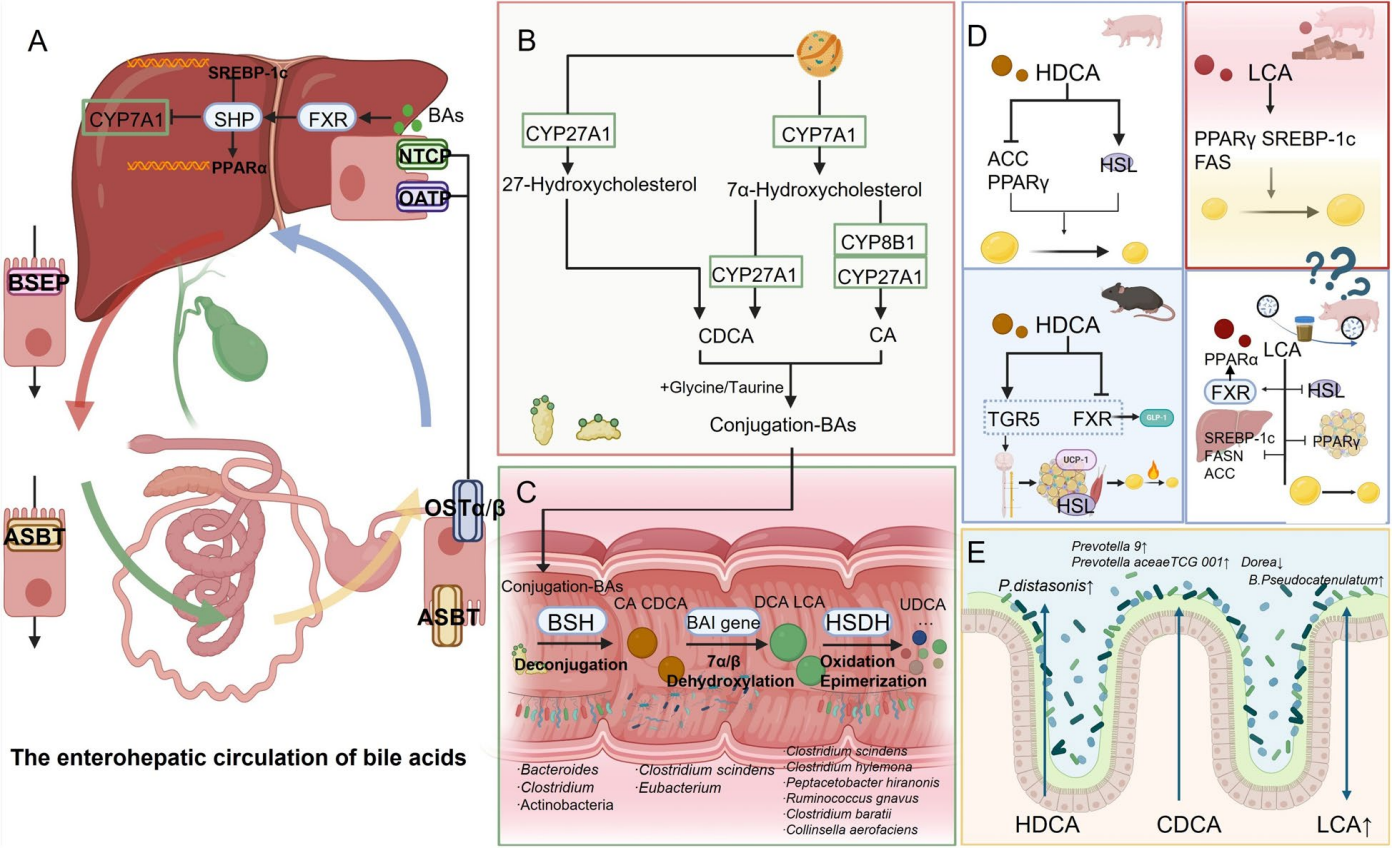
The underlying mechanisms of bile acids in the microbiota–gut–liver axis regulating fat deposition. **A** The enterohepatic circulation of bile acids. **B** Hepatic synthesis of conjugated bile acids. **C** Microbial biotransformation into secondary bile acids. **D** Bile acid-mediated mechanisms regulating fat deposition. **E** Reciprocal interactions between gut microbiota and bile acids. FXR, farnesoid X receptor; TGR5, Takeda G protein–coupled receptor 5; SHP, small heterodimer partner; PPARα, peroxisome proliferator-activated receptor alpha; ASBT, apical sodium-dependent bile acid transporter; BSEP, bile salt export pump; NTCP, sodium taurocholate cotransporting polypeptide; OATP, organic anion transporting polypeptide; CYP, cytochrome P450; HSDH, hydroxysteroid dehydrogenase; BSH, bile salt hydrolase; CA, cholic acid; CDCA, chenodeoxycholic acid; LCA, lithocholic acid; DCA, deoxycholic acid; HDCA, hyodeoxycholic acid; UDCA, ursodeoxycholic acid; *BAI* gene, bile acid–inducible gene

This emerging concept of a microbiota–gut–adipose axis emphasises that the intestine is not merely a passive conduit but an active metabolic and endocrine organ linking the microbiota to peripheral tissues. The structural integrity of the intestinal epithelium, its ability to absorb and modify bile acids, and its secretion of endocrine signals such as ANGPTL4 collectively mediate the cross-talk between gut microbiota and adipose depots in pigs and poultry. Understanding these mechanisms will enable more targeted microbiota-oriented strategies to modulate fat deposition in livestock (Fig. 3).

## Microbiota–gut–brain axis regulation of fat deposition

The microbiota–gut–brain axis (MGBA) is a complex network integrating neuronal, endocrine, and immune signals, which has emerged as a key regulator of energy homeostasis and fat deposition (human) [148, 149]. To improve clarity, the discussion below is organized by metabolite origin: fiber-derived metabolites (SCFAs) and amino acid-derived metabolites (tryptophan pathways).

### Fiber-derived metabolites: short-chain fatty acids

SCFAs produced by gut bacteria activate free fatty acid receptors GPR41 and GPR43 on enteroendocrine cells, thereby stimulating vagal afferent signaling to the hypothalamus and promoting insulin secretion. Concurrently, microbiota-derived acetylcholine enters the circulation and stimulates muscarinic M3 receptors on pancreatic β-cells, further augmenting insulin release. Insulin, in turn, activates the PI3K/Akt pathway, upregulates SREBP-1 to induce transcription of lipogenic enzymes (FASN and ACC), and promotes de novo fatty acid synthesis, while suppressing HSL to reduce lipolysis [150].

When SCFA production is reduced, lipid homeostasis is disrupted. Specifically, reduced SCFAs lead to decreased ANGPTL4 release and diminished AMPK activation in adipose tissue, promoting lipid accumulation and elevating circulating cholesterol. Reduced activation of GPR41 and GPR43 also lowers secretion of satiety hormones GLP-1 and peptide YY (PYY), resulting in decreased satiety, reduced energy expenditure, and increased adiposity [151]. Moreover, feeding behavior—meal frequency, portion size, and timing—profoundly influences energy intake and fat deposition. Notably, decreasing meal frequency has been shown to reduce fat accumulation and improve feed conversion efficiency in pigs [152].

Gut bacteria ferment dietary fibers and polyphenols to produce acetate, propionate, and butyrate. These SCFAs engage GPR41 (FFAR3) and GPR43 (FFAR2) on intestinal L-cells, stimulating GLP-1 and PYY release and conveying satiety signals to the brain. Butyrate can cross the blood–brain barrier and act directly on hypothalamic POMC neurons, suppressing orexigenic peptides such as AgRP and NPY to enhance satiety [41]. Although demonstrated in rodent models, these satiety-regulating effects provide mechanistic support for microbiota-based feeding interventions in pigs [153]. Collectively, SCFAs link fiber fermentation with reduced feed intake and improved lipid balance, providing a foundation for dietary interventions in swine.

### Amino acid-derived metabolites: tryptophan pathways

Tryptophan metabolism by gut microbes generates multiple metabolites—indole derivatives, kynurenine metabolites, and serotonin—that exert distinct effects on feeding and lipid metabolism.

#### Indole-3-lactic acid (ILA)

Time-restricted feeding (eTRF) in pigs markedly increases the abundance of *Ligilactobacillus salivarius*, *L. animalis*, and *Limosilactobacillus mucosae*. These taxa convert tryptophan to ILA, which upregulates Math1 and Ngn3, suppresses Notch signaling, and promotes differentiation of intestinal stem cells into GLP-1–secreting enteroendocrine cells (EECs). Consequently, systemic and intestinal GLP-1 levels rise, acting on the hypothalamus and crossing the blood–brain barrier to inhibit dopaminergic reward pathways via DRD1, DRD5, HTR1B, and HTR4, thereby reducing feeding frequency and overall intake [154]. Importantly, production trials confirm that TRF/eTRF not only modifies gut microbiota and elevates ILA but also improves feed-to-gain ratio and reduces total feed intake without compromising body weight gain, linking microbial activity to practical performance outcomes [154]. Additional studies in growing pigs show that TRF reduces hepatic fat accumulation and downregulates lipogenic gene expression, consistent with microbial shifts toward *Lactobacillus* enrichment [155]. Furthermore, adjusting feeding frequency in finishing pigs alters cecal microbiota and bile acid spectra, significantly reducing backfat thickness and abdominal fat weight while improving feed efficiency and longissimus muscle area [156]. These findings highlight that microbial regulation of tryptophan metabolism is directly connected to carcass composition and economic traits in swine production.

#### Kynurenic acid (KYNA)

In contrast, microbiota-derived KYNA has been identified as a local modulator of feeding. Although KYNA cannot traverse the blood–brain barrier, it binds to GPR35 on vagal nerve endings, inhibiting nodose ganglion activity and attenuating excitatory input to the nucleus tractus solitarius (NTS). This disinhibition activates AgRP neurons in the arcuate nucleus, leading to NPY release and promoting acute feeding behavior [157].

#### Serotonin (5-HT)

A third branch directs tryptophan toward serotonin. In antibiotic-treated piglets, *Lactobacillus amylovorus* abundance increases, elevating acetate production that activates FFAR3 to upregulate TPH1 while suppressing IDO1, thereby promoting serotonin synthesis. Meanwhile, *Streptococcus alactolyticus* harbors *trpA* and *trpB*, increasing luminal tryptophan availability and fueling further 5-HT production [158]. Tryptophan-derived 5-HT influences hypothalamic control of feeding by downregulating 5-HT1B receptors, reducing serotonergic inhibition of AgRP/NPY neurons. This increases AgRP expression, decreases anorexigenic markers (POMC, CART, and MC4R), and activates AMPK, while suppressing mTOR/S6K1 signaling, collectively promoting feeding. Additionally, duodenal and serum levels of PYY decline, further weakening satiety signals [159]. Chronic activation of this pathway enhances lipid synthesis and adiposity.

### Integration and production relevance

Together, these findings illustrate that the metabolic fate of tryptophan is shaped by microbial composition, host physiology, and feeding regimen. TRF promotes the ILA–GLP-1 pathway, improving feed efficiency and reducing fat deposition, whereas dysbiosis or alternative dietary patterns may bias tryptophan flux toward KYNA or 5-HT, promoting adiposity.

**Fig. 4:**
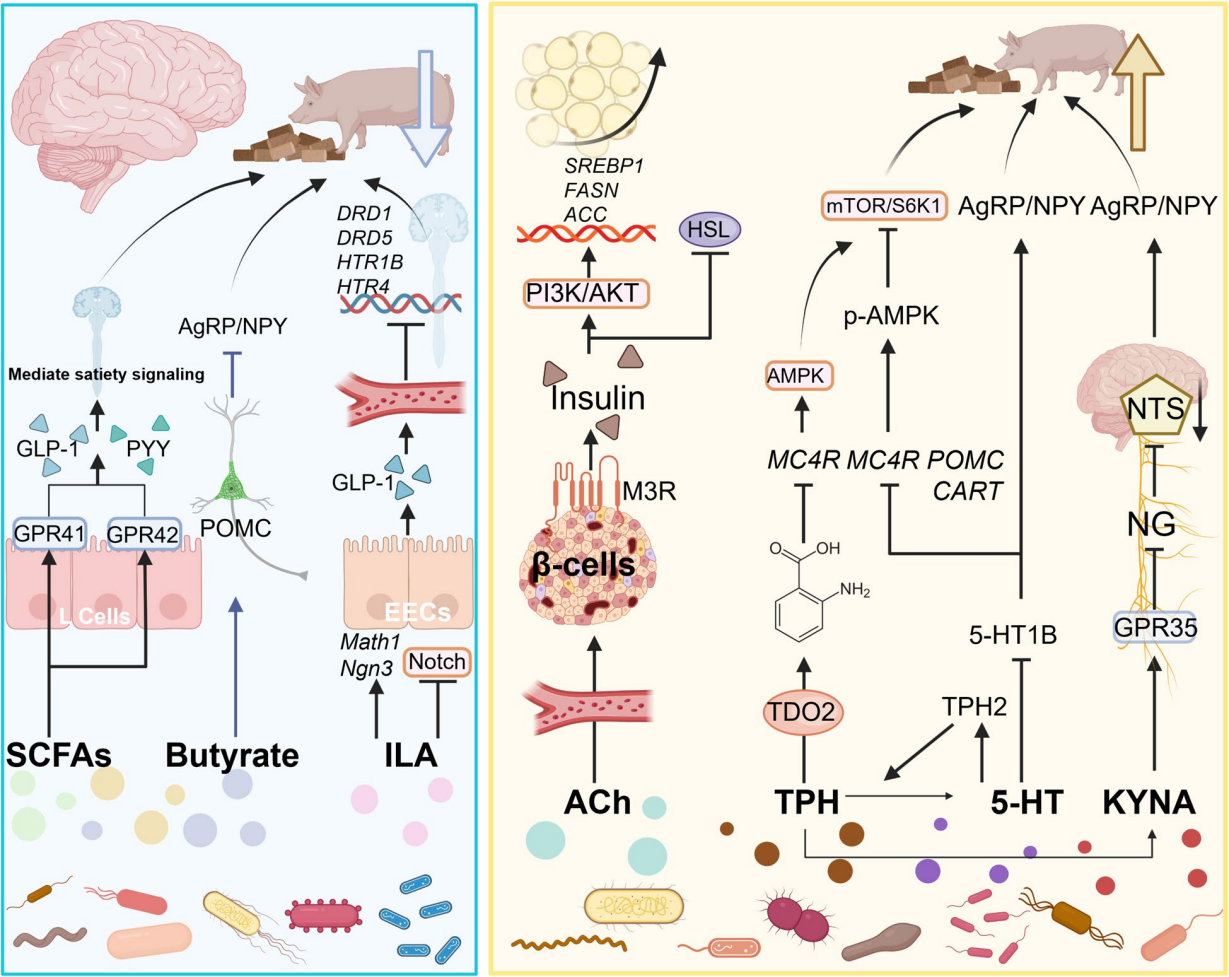
The underlying mechanisms of gut microbiota-derived metabolites modulating feeding behavior in swine. Blue shaded regions depict mechanisms that suppress feeding behavior, primarily by conveying satiety signals, inhibiting AgRP/NPY neuron activity, and downregulating corresponding receptor gene expression to enhance fullness and reduce appetite. Yellow shaded regions illustrate pathways that promote feeding behavior, mainly through activation of specific neural circuits and signaling pathways. ILA, indole-3-lactic acid; ACh, acetylcholine; 5-HT, 5-hydroxytryptamine (serotonin); KYNA, kynurenic acid; GPR41/42, G-protein coupled receptor 41/42; PYY, peptide YY; POMC, pro-opiomelanocortin; AgRP, agouti-related peptide; NPY, neuropeptide Y; CART, cocaine- and amphetamine-regulated transcript; MC4R, melanocortin 4 receptor; M3R, muscarinic acetylcholine receptor 3; NTS, nucleus tractus solitarii; NG, nodose ganglion; PI3K/AKT, phosphoinositide 3-kinase/protein kinase B pathway; mTOR/S6K1, mechanistic target of rapamycin/ribosomal protein S6 kinase beta-1; p-AMPK, phosphorylated AMP-activated protein kinase; TPH, tryptophan hydroxylase; TPH2, tryptophan hydroxylase 2; TDO2, tryptophan 2,3-dioxygenase

Understanding this intricate signaling network opens avenues for precision feeding and microbial interventions—such as probiotics, synbiotics, or controlled feeding regimens—to optimize backfat thickness, increase lean meat percentage, and enhance economic efficiency in swine production (Table 3 and Fig. 4).

**Table 3:** Gut microbiota-derived signaling molecules and their physiological functions

Signaling molecule	Source/production mechanism	Primary targets/pathways	Effect on fat deposition	References
Short‐chain fatty acids (SCFAs)	Fermentation of dietary fiber and polyphenols by gut microbiota	Activates GPR41 (FFAR3) and GPR43 (FFAR2) on intestinal L cells, stimulating GLP‐1 and PYY secretion; butyrate can cross the blood–brain barrier to act on hypothalamic POMC neurons, inhibiting AgRP/NPY	High SCFA levels enhance satiety and reduce food intake, thereby decreasing fat deposition; low SCFA levels promote lipid storage and increase serum cholesterol, leading to increased fat deposition	[41, 151]
Acetylcholine	Metabolite of gut microbiota	Activates M3 muscarinic receptors on pancreatic β cells via circulation, promoting insulin release	Indirectly promotes lipogenesis by enhancing insulin secretion	[150]
Indole‐3‐lactic acid (ILA)	Tryptophan metabolite produced by bacteria such as *Ligilactobacillus*	Upregulates transcription factors Math1 and Ngn3, inhibits Notch signaling, and promotes differentiation of intestinal stem cells into GLP‐1–secreting enteroendocrine cells (EECs)	By increasing GLP‐1 secretion, suppresses appetite, reduces food intake, and thereby decreases fat deposition	[154]
Kynurenic acid (KYNA)	Tryptophan metabolite of gut microbiota	Binds to GPR35 on gut vagal nerve endings, inhibits nodose ganglion (NG) activity, reduces excitatory input to the nucleus tractus solitarius (NTS), disinhibits ARC AgRP neurons, and promotes NPY release	Increases short‐term feeding behavior, potentially promoting fat deposition	[157]
Tryptophan	Synthesized by bacteria such as *Streptococcus alactolyticus*	Activates hypothalamic KYNA and 5‐HT metabolic pathways to regulate feeding	Significantly increases feed intake in pigs, promotes fat deposition	[159]
Serotonin (5‐HT)	Tryptophan conversion mediated by bacteria such as *Lactobacillus amylavorans*	Downregulates 5‐HT1B receptor expression, reducing inhibitory signaling on NPY/AgRP neurons	Significantly increases feed intake in pigs, promotes fat deposition	[159]

## Prospects for livestock and poultry applications

Building on animal evidence summarized above, microbiota-targeted strategies show practical promise for simultaneously improving pork quality (greater, better-partitioned marbling) and feed efficiency in production settings. In pigs, leveraging fiber-driven SCFAs and the bile acid–FXR/TGR5 axis provides tractable levers: higher SCFA signaling links to reduced feed intake and improved lipid balance, offering a mechanistic basis for dietary/prebiotic interventions in finishing herds.

Probiotic candidates already supported by livestock data include *Lactobacillus delbrueckii* (in pigs), which improves lipid handling via fecal bile acid excretion and AMPK-linked suppression of lipogenesis, and thus is a rational tool to trim visceral and subcutaneous fat while sustaining growth [42]. In parallel, *Clostridium butyricum* has been associated with enhanced carbohydrate fermentation and improved feed efficiency in pigs, suggesting value as a core component in synbiotic blends [25]. Precision feeding offers a management-friendly route: time-restricted feeding (TRF/eTRF) in pigs reshapes *Lactobacillus*–indole-3-lactic acid–GLP-1 signaling, improves feed-to-gain, reduces intake without sacrificing growth, and—when feeding frequency is optimized in finishers—reduces backfat and improves feed efficiency, aligning carcass outcomes with economic goals [154, 155].

For meat quality, microbiota selection can steer marbling independent of excessive subcutaneous fat: fecal microbiota transplantation from high-IMF Laiwu donors increased intramuscular fat yet reduced backfat in Duroc recipients [46].

Poultry data similarly support deployability: transferring cecal microbiota from lean donors lowers abdominal fat, and the chicken cecal microbiome’s modulation of bile acid/riboflavin metabolism correlates with breast IMF—highlighting targets for probiotic or feed additive design [47, 147]. Notably, because excess visceral fat directly impairs feed efficiency in pigs, prioritizing consortia/diets that curb VAT (e.g., via SCFA production or BA remodeling) should yield immediate FCR benefits while supporting lean gain [37].

Together, these animal-based lines of evidence justify near-term field trials that combine (i) targeted probiotics (e.g., *C. butyricum* and *L. delbrueckii*), (ii) prebiotic substrates to boost SCFAs, and (iii) phase-specific feeding regimens (TRF/eTRF) to tune MGBA and gut–liver signaling toward lower backfat, higher IMF, and superior feed conversion in pigs, with parallel strategies applicable to broilers. Nevertheless, translating these approaches from controlled experiments to large-scale commercial farms may face challenges such as cost, operational complexity, and environmental variability; addressing these issues will be essential for successful industry adoption.

## Conclusions

Recent studies have revealed the essential role of gut microbiota in regulating host lipid metabolism through microbial metabolites, bile acid signaling, and neuroendocrine pathways. However, several knowledge gaps remain. First, most current research focuses on single taxa or isolated mechanisms, whereas the complex, multi-organ interactions within the microbiota–gut–liver–brain–adipose axis are not yet fully understood. Future work should integrate multi-omics approaches (metagenomics, transcriptomics, metabolomics) to systematically dissect microbial network functions and metabolite–receptor interactions. Second, the majority of evidence derives from rodent or in vitro models, and more studies in pigs and poultry are urgently needed to establish direct links between microbial regulation and production traits such as feed efficiency, carcass composition, and intramuscular fat deposition. Third, translation into practice remains limited: although probiotics (e.g., *Clostridium butyricum* and *Lactobacillus delbrueckii*), prebiotics, and precision feeding strategies (e.g., time-restricted feeding) show promise, their stability, cost-effectiveness, and scalability under farm conditions require rigorous field trials. Addressing these challenges will be critical to move from proof-of-concept toward industry-level applications. Ultimately, a deeper mechanistic understanding combined with translational studies will accelerate the development of microbiota-targeted strategies to optimize fat deposition, improve pork and poultry meat quality, and enhance feed efficiency in commercial animal production.

## Data Availability

Not applicable.

## Abbreviations

ACC: Acetyl-CoA carboxylase
AhR: Aryl hydrocarbon receptor
AMPK: AMP-activated protein kinase
ANGPTL4: Angiopoietin-like 4
AgRP: Agouti-related peptide
BAs: Bile acids
BSHs: Bile salt hydrolases
CA: Cholic acid
cAMP: Cyclic adenosine monophosphate
CDCA: Chenodeoxycholic acid
CoA: Coenzyme A
DCA: Deoxycholic acid
EECs: Enteroendocrine cells
FAS: Fatty acid synthase
GPCR: G protein-coupled receptor
HDAC: Histone deacetylase
HDCA: Hyodeoxycholic acid
HSL: Hormone-sensitive lipase
ILA: Indole-3-lactic acid
IMF: Intramuscular fat
KYNA: Kynurenic acid
LDA: Linear discriminant analysis
LEfSe: Linear discriminant analysis effect size
LPL: Lipoprotein lipase
LPS: Lipopolysaccharide
MAPK: Mitogen-activated protein kinase
MCT1: Monocarboxylate transporter 1
MGBA: Microbiota–gut–brain axis
mNGS: Metagenomic next-generation sequencing
NPY: Neuropeptide Y
POMC: Proopiomelanocortin
PPARα: Peroxisome proliferator-activated receptor alpha
PPARγ: Peroxisome proliferator-activated receptor gamma
PYY: Peptide YY
SAT: Subcutaneous adipose tissue
SBAs: Secondary bile acids
SCFAs: Short-chain fatty acids
SHP: Small heterodimer partner
SMCT1: Sodium-coupled monocarboxylate transporter 1
SREBP1: Sterol regulatory element-binding protein 1
VAT: Visceral adipose tissue
